# Estimating radiation effective doses from whole body computed tomography scans based on U.S. soldier patient height and weight

**DOI:** 10.1186/1471-2342-11-20

**Published:** 2011-10-17

**Authors:** Robert D Prins, Raymond H Thornton, C Ross Schmidtlein, Brian Quinn, Hung Ching, Lawrence T Dauer

**Affiliations:** 1 Department of Medical Physics, Memorial Sloan-Kettering Cancer Center, 1275 York Ave. New York, NY 10021, USA; 2 Department of Environmental Health Sciences, Mailman School of Public Health, Columbia University, 722 West 168th Street, New York, NY 10032, USA

## Abstract

**Background:**

The purpose of this study is to explore how a patient's height and weight can be used to predict the effective dose to a reference phantom with similar height and weight from a chest abdomen pelvis computed tomography scan when machine-based parameters are unknown. Since machine-based scanning parameters can be misplaced or lost, a predictive model will enable the medical professional to quantify a patient's cumulative radiation dose.

**Methods:**

One hundred mathematical phantoms of varying heights and weights were defined within an x-ray Monte Carlo based software code in order to calculate organ absorbed doses and effective doses from a chest abdomen pelvis scan. Regression analysis was used to develop an effective dose predictive model. The regression model was experimentally verified using anthropomorphic phantoms and validated against a real patient population.

**Results:**

Estimates of the effective doses as calculated by the predictive model were within 10% of the estimates of the effective doses using experimentally measured absorbed doses within the anthropomorphic phantoms. Comparisons of the patient population effective doses show that the predictive model is within 33% of current methods of estimating effective dose using machine-based parameters.

**Conclusions:**

A patient's height and weight can be used to estimate the effective dose from a chest abdomen pelvis computed tomography scan. The presented predictive model can be used interchangeably with current effective dose estimating techniques that rely on computed tomography machine-based techniques.

## Background

This research was driven by the need to estimate the radiation dose from computed tomography scans given to soldiers and civilians injured in austere environments. Nearly 50% of all injuries to United States Army soldiers in Operation Iraqi Freedom and Operation Enduring Freedom occurred in the head/neck, abdomen, and thorax region of the body [1,2]. Injuries in these regions are most commonly assessed using diagnostic x-ray scanning modalities such as computed tomography (CT). Current CT machines are able to scan an entire body in as little as 30 seconds making them particularly advantageous for diagnosing the extent of injuries sustained in traumatic events [3]. As a result, CT scanning is an integral part of the medical treatment schema from patient initial diagnosis through rehabilitation. In fact, because of this, CT scanning usage has steadily increased since its inception in 1972 [4]. While CT scanners have traditionally been used in fixed large hospital facilities technological advancements have made deployable CT machines a reality on battlefield environments [5].

While CT imaging as many advantages over other diagnostic modalities for diagnosing trauma injuries, it does have some drawbacks. CT procedures give patients more radiation dose than traditional x-ray imaging modalities. Because of this and CT's increased use, patients are exposed to more dose which may result in unintended health effects. As such, trade-offs exist between risk and benefit in the use of CT. In order to manage the risks associated with CT effectively, healthcare providers need to be able to estimate and track the dose these patients receive from their CT scan(s). Additionally, CT generates an order of magnitude more information than traditional medical imaging modalities.

The Joint Patient Tracking Application was developed to allow users to get real-time information on the status of their injured troops [6]. However, the trauma record does not show how many radiographic procedures were performed in the trauma diagnosis. Furthermore, advances in diagnostic imaging have not made the management of patient imaging records easier. This is illustrated by the fact that for a brief time period, one combat hospital switched from film radiography to digital radiography and then back to film radiography because of increased throughput during trauma situations and the inability of outside facilities to read the compact disks with the films saved on them [7].

The probability of repeat diagnostic scanning procedures increases when failure to transfer relevant understandable radiographic information along with the patient occurs. Additionally, as the level of integrated hospital care increases, the radiographic technology also increases resulting in multiple diagnostic procedures on the same area with different diagnostic modalities. Increasing numbers of radiographic procedures are not unique to military medicine. Victims of trauma receive multiple diagnostic scans and thus are at an increased risk of detrimental health risks from cumulative radiation dose [3,8,9].

Quantification of the risk derived from the repeated use of CT scans is needed in order to assess the consequences of the increased dose to these soldiers. A common metric for estimating the dose from CT scans, effective dose, describes the relationship between the probability of stochastic effects from radiation and equivalent dose for a mathematical reference phantom [10]. Estimating the effective dose is usually performed using machine-based parameters and derived conversion coefficients [4,11].

However, if the CT machine-based parameters for the scan are unknown because the requisite information is lost as a patient is transferred from one hospital to another, the only other means of estimating the effective dose from a CT scan is through the use of broad estimates based on published nominal values [12]. We propose an alternate method that estimates the chest abdomen pelvis scan effective dose to a mathematical phantom based upon the patient's height and weight at the time of the scan.

## Methods

The approach we took to develop the alternate method for estimating effective dose involved three steps. The first step required the development of a mathematical model based upon a fictitious population of phantoms representing a range of body parameters common for U.S. Army soldiers. The second step verified the model by comparison of absorbed doses to organs using anthropomorphic phantoms. Finally, the third step validated the model with a real patient dataset. The development of estimating the effective dose model differs from the current method of estimation which uses machine-based parameters.

The most common method of estimating the effective dose when machine-based parameters from a CT scan are available involves multiplying the dose-length-product (DLP), a product of the volume computed tomography dose index (CTDI_vol_) and the scanning length, by a conversion coefficient. The DLP is available by referencing the dose report generated by most commercial scanners at the end of the CT scanning procedure. Conversion coefficients have been derived using Monte Carlo simulations and experimental measurements.

Commercial CT scanners provide an estimate of the DLP in the dose report generated at the end of a scanning procedure. The DLP is derived from the CTDI_vol _which is measured using a 32-cm diameter acrylic cylinder and a 100-mm long pencil shaped ionization chamber [13]. The chamber provides the dosimetry measurements at the center of the cylinder (c) and on the periphery (p) of the cylinder from which the weighted CTDI (CTDI_w_) is calculated using

(1) $$\text{CTD}{\text{I}}_{\text{w}}=\frac{2}{3}\text{CTD}{\text{I}}_{\text{p}}+\frac{1}{3}\text{CTD}{\text{I}}_{\text{c}}.$$

CTDI_vol _is calculated by considering both the CTDI_w _and the pitch of the machine given by

(2) $$\text{CTD}{\text{I}}_{\text{vol}}=\frac{\text{CTD}{\text{I}}_{\text{w}}}{\text{pitch}},$$

where pitch is the table increment travelled per complete rotation of the x-ray tube. DLP is the product of the CTDI_vol _and the scan length.

(3) $$\text{DLP = CTD}{\text{I}}_{\text{vol}}\times \text{scan length}$$

Effective dose, the primary outcome measure of our study, can be calculated from the CT machine-based parameters and is the product of the DLP (mGy cm) and specific conversion coefficients (CC) (mSv mGy^-1 ^cm^-1^), using

(4) E=DLP×CC

Conversion coefficients are available in several publications including the American Association of Physicists in Medicine (AAPM) Report 96 [11] and the National Council on Radiation Protection and Measurements (NCRP) Report 160 [4]. The conversion coefficient used in our study for a chest abdomen pelvis (CAP) scan is 0.015 mSv mGy^-1 ^cm^-1^[14].

The radiation dose from axial scanning is converted to radiation dose from helical scanning by dividing the axial scanning dose by the CT pitch.

We used a commercially available software program, (PCXMC 2.0.1 (Personal Computing X-ray Monte Carlo), STUK, Finland) [15], for calculating patient average absorbed organ doses in medical x-ray examinations. The software uses mathematical hermaphrodite phantoms.

As the software is primarily designed for projection radiography, four projections (anterior posterior (AP), posterior anterior (PA), right lateral (RLAT), and left lateral (LLAT)) were used to simulate a 360 degree exposure from a CT machine [16]. Each projection had the same slice thickness as a CT axial slice. Calibration factors were developed for effective dose comparison with a commonly used CT software program (ImPACT CT Patient Dosimetry Calculator, version 0.99x; ImPACT, London, England).

Mathematical phantoms of varying sizes were developed to represent the range of sizes of U.S. Army soldiers. Typical-sized U.S. Army soldiers have a body mass index (BMI, kg/m^2^) of 20 to 30 [17,18]. Development of the phantom population used BMI, ranging from 18 (underweight) to 36 (obese), as a means of determining the height and weight of a theoretical U.S. Army population. Corresponding heights and weights were calculated from the BMI values. One hundred hermaphrodite mathematical phantoms with height and weight ranging from 5 feet 1 inch up to 6 feet 7 inches and 95 pounds up to 317 pounds were configured for use within the software. The methodology for determining the number of phantoms of varying height and weight was based on common guidelines that suggest the use of ten observations for each predictor [19,20].

In our study, all dose calculations per slice were simulated with 1 × 10^6 ^photons. Anode angle was set at 7 degrees with a beam quality half-value layer of 7.4 mm aluminum [21]. X-ray tube voltage was set at 120 kV for all calculations and software-required entrance air kerma values were obtained from measurements of the four projections at the center point of a typical chest abdomen pelvis scan using an anthropomorphic phantom. Averaged absorbed doses to organs were generated and recorded.

Multivariate regression analysis was performed using the R Project for Statistical Computing statistical program [22] in order to determine a best-fit model using height and weight as predictors of the effective dose. Full regression models were generated that identified effective dose as the dependent variable and the independent variables as height (cm), height^2 ^(cm^2^), weight (kg), and weight^2 ^(kg^2^). Bayesian information criterion techniques were used for variable selection. Statistical significance was defined as p < 0.05.

**Figure 1:**
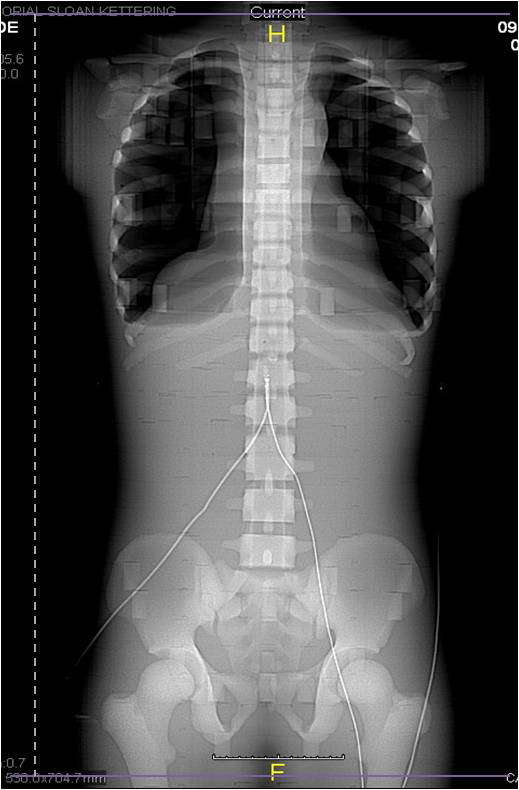
**Representative Chest Abdomen Pelvis Scanning Range**. Representative scanning range (vertical dashed line) of a chest abdomen pelvis (CAP) scan.

Experimental verification measurements were performed using two adult anthropomorphic phantoms (CIRS, Inc., Norfolk, Virginia). The female adult anthropomorphic phantom (height 160 cm and weight 55 kg) and male adult anthropomorphic phantom (height 173 cm and weight 73 kg) were manufactured with dosimetry verification plugs enlarged to accommodate optically stimulated luminescent dosimeters utilizing an Al_2_O_3 _detector (Landauer Nanodot, Landauer, Inc., Glenwood, IL). All computed tomography scans were performed using a GE LightSpeed 16 (General Electric Healthcare, Waukesha, WI) (Figure 1). Manufacturer recommended calibration procedures were followed prior to irradiation [23]. For the purposes of this study, an 80 kVp calibration set was used and final dosimeter readings were adjusted to account for CT scanning (at 120 kVp) by multiplying each final reading by 1.15 based upon an experimentally determined energy response curve corresponding to the manufacturer calibration and usage instructions.

Both phantoms were scanned with the same experimental technique factors: 120 kVp, automatic mA (220 - 380 mA), slice thickness of 10-mm, and a helical scanning pitch 1.375.

The model for estimating effective dose presented in this paper was compared to the method of estimating effective dose using DLP and conversion coefficients because this latter method is the current accepted method [11,24]. An institutional review board approved retrospective study (Memorial Sloan-Kettering Cancer Center WA 0313-10 dated 23 June 2010) (n = 28) was conducted to compare the developed height weight predictive effective dose model with the current effective dose estimation method of multiplying the DLP by a published conversion coefficient. A Bland-Altman plot was used to visually inspect the variation between the two methods of estimating the effective dose. Bland-Altman plots (also known as an average versus difference plot) show the average of the methods on the x-axis and the difference between the methods on the y-axis [25]. This type of plot is effective in visually displaying the potential for systematic differences and method agreements.

## Results

In the univariate analysis, patient height (H) (cm) and weight (W) (kg) demonstrated a significant association with effective dose (E) (mSv) and were entered into the multivariable linear regression analysis (equation 5). The variation (R^2 ^= 0.96) in the effective dose is primarily explained by a patient's height and weight. In the multivariable linear regression analysis, increasing patient height had a positive effect on the effective dose whereas increasing patient weight had a negative effect on the effective dose. A 1-cm increase in a patient's height is associated with a 0.3% increase in the effective dose. Similarly, a 1-kg increase in a patient's weight is associated with a 0.5% decrease in the effective dose given by

(5) E (mSv)=18+0.067 H(cm)-0.11 W (kg).

Since effective dose is not a measurable quantity in and of itself, verification of the model (equation 5) was conducted by comparing experimentally obtained organ doses for eleven different organs which represent important components of effective dose to the organs doses estimated using the Monte Carlo software code. All of the organs listed in the tables (tables 1 and 2) are included within the primary scanning field of view except the brain and eyes.

**Table 1:** Organ absorbed doses

Organ	Female			Male		
	
	Measured Absorbed Dose (mGy)	Calculated Absorbed Dose (mGy)	ABS % Difference	Measured Absorbed Dose (mGy)	Calculated Absorbed Dose (mGy)	ABS % Difference
Adrenals/Gall Bladder	26.90 ± 0.03	25 ± 1	2	27.9 ± 0.4	23 ± 1	5
Brain	0.29 ± 0.02	0.4 ± 0.1	8	0.7 ± 0.3	0.4 ± 0.1	14
Colon	22.3 ± 0.7	21.1 ± 0.8	1	28 ± 5	20 ± 0.8	8
Esophagus	25 ± 1	23 ± 1	2	22.1 ± 0.4	21 ± 1	1
Eye	0.495 ± 0.008	NA		0.74 ± 0.08	NA	
Kidney	24.2 ± 0.5	31.5 ± 0.9	7	24.2 ± 0.8	30.3 ± 0.9	5
Liver	26.0 ± 0.2	29.5 ± 0.3	3	26.3 ± 0.9	28.1 ± 0.3	2
Lung	27 ± 1	33.9 ± 0.7	6	24 ± 1	32.4 ± 0.6	7
Pancreas	25.9 ± 0.4	25.1 ± 0.7	1	28.5 ± 0.2	23.2 ± 0.7	5
Thymus	23.5 ± 0.3	25 ± 5	2	27.0 ± 0.5	24 ± 5	3
Thyroid	12.3 ± 0.4	24 ± 9	16	30.2 ± 0.8	24 ± 9	6
Uterus/Testes	25.6 ± 0.1	21 ± 4	5	8 ± 4	0.8 ± 0.4	41

Organ absorbed doses for a chest abdomen pelvis scan. The measured absorbed dose values are from the anthropomorphic phantoms and the calculated absorbed dose values are from the mathematical phantom.

**Table 2:** Estimates of the effective dose

Organ	ICRP103 Tissue Weighting Factor	Measured Equivalent Dose (Female) (mSv)	Calculated Equivalent Dose (mSv)	ABS Diff (%)	Measured Equivalent Dose (Male) (mSv)	Calculated Equivalent Dose (mSv)	ABS Diff (%)
Brain	0.01	0.0029 ± 0.0002	0.004 ± 0.001		0.007 ± 0.003	0.004 ± 0.001	
Colon	0.12	2.68 ± 0.08	2.5 ± 0.1		3.4 ± 0.6	2.4 ± 0.1	
Esophagus	0.04	1.00 ± 0.04	0.92 ± 0.04		0.88 ± 0.02	0.84 ± 0.04	
Liver	0.04	1.040 ± 0.008	1.18 ± 0.01		1.05 ± 0.04	1.12 ± 0.01	
Lung	0.12	3.24 ± 0.12	4.07 ± 0.08		2.9 ± 0.1	3.89 ± 0.07	
Thymus	0.04	0.94 ± 0.01	1.0 ± 0.2		1.08 ± 0.02	1.0 ± 0.2	
Thyroid	0.04	0.49 ± 0.02	1.0 ± 0.4		1.21 ± 0.03	1.0 ± 0.4	
Remainder	0.12	3.08 ± 0.08	3.1 ± 0.5		2.7 ± 0.5	2.3 ± 0.2	
Organs:							
Pancreas							
Uterus							
Kidney							
Adrenals/Gall							
Bladder							

Effective Dose (mSv)		12.5 ± 0.2	13.7 ± 0.7	9%	13.1 ± 0.8	12.5 ± 0.6	5%

Effective dose for a chest abdomen pelvis scan.

The percent difference between the effective doses determined using either the mathematical phantom or experimental measurement is 9% for the female anthropomorphic phantom and 5% for male anthropomorphic phantom.

**Figure 2:**
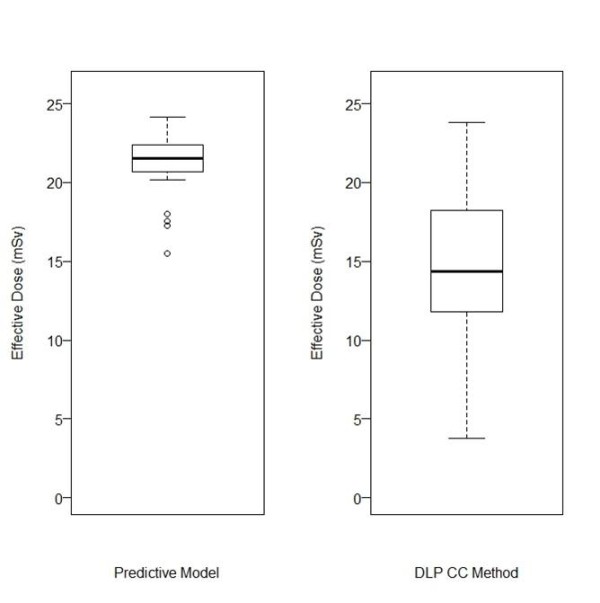
**Effective Dose Comparison**. Effective dose box-and-whisker plot comparison showing the two different methods of calculation.

The absolute percent difference of the average effective dose for the IRB-approved population (n = 28) for the two means of calculating the effective doses was 33%. The population average effective dose calculated using the predictive model was 21 ± 2 mSv, while the population average effective dose calculated using the DLP CC method was 15 ± 5 mSv (Figure 2). Three of the four effective dose outliers shown on the predictive model plot are due to the patient BMI being greater than the range of BMIs (18 - 36 kg m^-2^) used to develop the predictive model. The fourth effective dose outlier shown on the predictive model plot is due to the patient BMI (35 kg m^-2^) being at the upper range of the BMIs used to develop the predictive model. The two means of calculating the effective doses are significantly different (paired t-test, p < 0.001) so analyzing the average of the effective doses versus the difference of the effective doses was performed.

**Figure 3:**
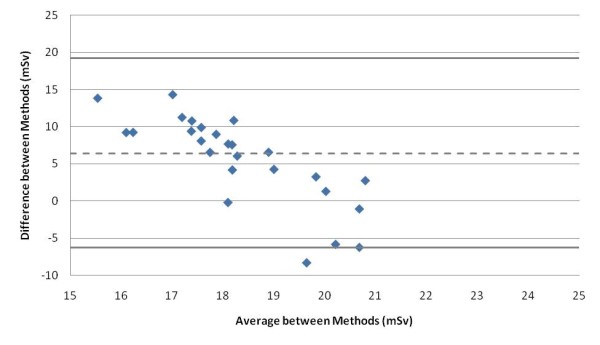
**Bland-Altman Analysis**. Bland-Altman analysis of the two effective dose calculation methods (predictive model and the DLP CC method). Y-axis difference values are calculated by subtracting the DLP CC values from the Predictive Model values.

**Figure 4:**
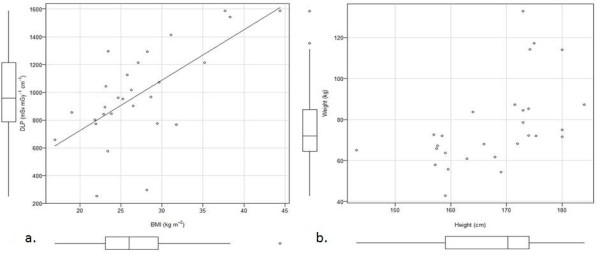
**Patient population data**. Patient population verification data. a) Scatter plot and box-and-whisker plot of the BMI values (median BMI = 26 kg m^-2^) range versus DLP range (median DLP = 956.19 mSv mGy^-1 ^cm^-1^). Least squares regression line shows the direct relationship between BMI and DLP. b) Scatter plot and box-and-whisker plot of the height (cm) (median height = 170.25 cm) versus weight (kg) (median weight = 72 kg).

Figure 3 shows the Bland-Altman plot for the two methods of calculating the effective dose from a chest abdomen pelvis scan. The height weight predictive model has a positive bias compared to the DLP CC method (mean of the differences = 6.38 mSv). The positive bias indicates that the height weight predictive model consistently estimated slightly higher effective doses than the DLP CC method. There is no significant systematic difference because the line of equality is within the ±1.96 standard deviation lines. All plotted values are within two standard deviations (solid heavy horizontal lines) of the mean (dashed horizontal line). Those plotted values that are near the -1.96 standard deviation line are a result of very large DLP values due to the patient BMI being greater than the BMIs used to develop the predictive model (Figure 4).

## Discussion

In cardiology scans, it is well known that a patient's BMI is correlated with the effective dose from computed tomography scans [26,27]. Since BMI is a quantity comprising height and weight, the development of a model based upon height and weight will allow for estimating effective dose with minimal information and will result in similar accuracy of effective dose estimates when compared to current methods using CT machine-based parameters and these parameters are unknown. There is currently no known research specifically addressing such a model for chest abdomen pelvis CT scans. This research was designed to address that information gap.

The goal of using height and weight as predictors was to obtain a minimally confounded estimate of the effect of patient dimensions on effective dose from CT trauma protocols. A physician can utilize the predictive model (equation 5) to estimate the effective dose for the CT whole body (chest abdomen pelvis) scan. Specific machine characteristics were not assessed as predictors because this information is often not known for retrospective assessment of effective dose from CT scans. The coefficient of determination, R^2^, was used to describe the variability in the calculated effective dose explained by the linear regression model.

Utilizing Bland-Altman [25,28] plots to compare two methods allow us to determine whether or not the two methods can be used interchangeably. Since both calculations are an estimate of the effective dose the limits of agreement were set to be +/- 2 standard deviations of mean difference of the two methods. Convention allows that when the difference between the two methods lies within two standard deviations of the difference mean, either method can be used with respect to accuracy [29]. The height weight predictive model is consistent with current literature which suggests that the DLP CC is known to underestimate the effective dose per scan [11,30-32]. Furthermore, literature suggests that experimental measurements of effective dose will often be higher than DLP CC methods for calculating effective dose because DLP CC methods can underestimate the effective dose by up to 37% [31-33]. Part of the reason for this difference is that conversion factors are highly dependent upon the specific size of the phantom used and the assumed scanning length [30].

DLPs can also underestimate the total energy imparted over the scanning length [11]. When scanning lengths are increased or decreased, organs are either brought into the scanning field-of-view or removed from the scanning field-of-view. For scanning regions which include fewer major organs (brain scan and cervical spine scan), differences between various methods of calculating effective doses result from dimensional differences between phantoms and patients. Dose variations between phantoms, physical and mathematical, will be less when organs are small in volume and can be easily represented by an averaged point dose estimate. Dose variations between phantoms will also be less for large organs which receive a fairly uniform absorbed dose throughout its volume [34].

This research was limited in the verification and validation of the model by using only two reference anthropomorphic phantoms and one type of CT scanner. Additional research should be performed verifying the model with other CT scanner types since dose variations among CT scanners made by different manufacturers is known to occur [35,36].

## Conclusions

The model described in this article can be used to estimate the effective dose from a chest abdomen pelvis CT scan to a mathematical phantom based on a patient's height and weight. Effective dose estimation gives the medical professional a means of comparing patient doses from CT with those of other radiation diagnostic modalities. This model allows for retrospective effective dose estimation when the machine parameters are not known for patient populations of similar characteristics with the mathematical and anthropomorphic phantoms.

## Competing interests

The authors declare that they have no competing interests.

## Authors' contributions

RDP and LTD conceived of the study, collected the bulk of the data, and drafted the manuscript. CRS, RHT, BQ, and HC participated in the design and coordination of the study and assisted in the collection of the data. All authors read and approved the final manuscript.

## Author information

RDP: Lieutenant Colonel Robert D. Prins, United States Army, completed his doctoral program at Columbia University and performed his doctoral research at Memorial Sloan-Kettering Cancer Center.

## Pre-publication history

The pre-publication history for this paper can be accessed here:

http://www.biomedcentral.com/1471-2342/11/20/prepub
